# Evaluation of Kinetic Models for the Catalytic Hydrogenation of Levulinic Acid to γ-Valerolactone over Nickel Catalyst Supported by Titania

**DOI:** 10.3390/molecules30071400

**Published:** 2025-03-21

**Authors:** Carlos Alberto Sepulveda Lanziano, Cristiane Barbieri Rodella, Reginaldo Guirardello

**Affiliations:** 1 School of Chemical Engineering, University of Campinas, Av. Albert Einstein n. 500, Campinas 13083-852, Brazil; 2 Brazilian Synchrotron Light Laboratory (LNLS), Brazilian Center for Research in Energy and Materials (CNPEM), R. Giuseppe Máximo Scolfaro n. 10000, Campinas 13083-100, Brazil

**Keywords:** kinetic study, γ-valerolactone, levulinic acid, hydrogenation, heterogeneous catalysis, nickel, titania, biomass conversion

## Abstract

The search for alternative sources of, and substitutes for, chemicals derived from fossil-based feedstocks encourages studies of heterogeneous catalysts to increase the feasibility of sustainable production of biomass derivatives, such as γ-valerolactone, among others. In this context, first, the performance of a titania-supported nickel catalyst (a non-noble catalyst) was evaluated in the reaction of hydrogenation of levulinic acid to γ-valerolactone in water using molecular hydrogen. The methods used included the synthesis of titania via the solgel method and nickel deposition by deposition–precipitation via removal of the complexing agent. The nickel was activated in a flow of hydrogen; the temperature of reduction and the calcination step were investigated with experiments at reaction conditions to study the catalyst’s stability. Then, after a statistical evaluation of several proposed kinetic models, the kinetics of the reaction was found to be best represented by a model obtained considering that the reaction over the surface was the determinant step, followed by the non-dissociative adsorption of hydrogen and the competitive adsorption among hydrogen, levulinic acid, and γ-valerolactone. With that model, the activation energy of the levulinic acid to 4-hydroxypentanoic acid step was (47.0 ± 1.2) kJ mol^−1^, since the determinant step was the hydrogenation reaction of the levulinic acid to 4-hydroxypentanoic acid. It was also concluded that the catalyst prepared was stable, active, and selective to γ-valerolactone.

## 1. Introduction

γ-valerolactone is one of the chemical products derived from lignocellulosic biomass with high applicability in the industry. Several studies have shown its potential to be used as a solvent, fuel, fuel precursor, and intermediate for other chemicals [1,2,3,4,5,6]. One of the routes for producing γ-valerolactone is the hydrogenation of levulinic acid, one of the platform molecules obtained from the processing of lignocellulosic biomass [6]. It is estimated [7] that levulinic acid could be used as a raw material to produce γ-valerolactone (and other products) once the market price of levulinic acid is below 1 USD/kg.

The first catalytic studies on the hydrogenation reaction of levulinic acid to γ-valerolactone were carried out mainly with catalysts based on noble metals (Ru, Pd, Au, Pt) [8,9,10,11]. These metals continue to be widely studied, especially ruthenium, which has shown high catalytic activity [6,12,13,14,15,16,17,18,19,20,21,22]. However, the cost of noble metals is one of the main reasons that encourage the study of catalysts based on base metals, such as nickel [6,23,24,25,26,27,28,29,30,31,32], copper [6,30,33,34], and cobalt [35,36,37].

Along with research on metals that favor the formation of γ-valerolactone, studies have been carried out on these metals on different supports, such as coal [9,10,11,15,25,31], titania [2,8,38,39], alumina [27,35,36,40], zirconia [2,41], and silica [2,23,36]. The support aims to facilitate the removal of the catalyst, increase the exposed active phase, improve stability, and/or participate in the catalytic cycle [8,9,23]. The latter is because the hydrogenation reaction of levulinic acid to γ-valerolactone has been shown to be favored by supports with acidic characteristics [17,21,23].

Another factor to consider in process development is solvent selection. From the point of view of green chemistry, there is a set of recommended substances [42] in which water is highlighted. These have been identified as advantageous solvents for organic reactions [42,43] due to their high availability and low cost, in addition to not having risks of toxicity and explosiveness, and that, a priori, their use does not affect the environment. In the case of the hydrogenation reaction of levulinic acid to γ-valerolactone, water is the most used solvent [2,8,9,10,11,12,16,21,33,34,40,41,44], especially when using noble metals. In the case of non-noble metals, in addition to water [33,34], there are studies without the use of solvent [23,24,25,27,28,30,36], generally carried out in the vapor phase, using dioxane [26,29,31,35] and alcohols [45,46].

In this context, nickel was chosen as the catalyst in this work, due to evidence in the literature that it is one of the non-noble metals with the best catalytic characteristics to the hydrogenation reaction of levulinic acid to γ-valerolactone [6,23,24,25,45,47,48]. Furthermore, there is an absence of studies related to models of the reaction kinetics of catalysts based on non-noble metals. So, in this work, several models were proposed and evaluated through a statistical analysis, to find the one that gives the best representation.

Water was defined as a solvent due to recommendations that show it to be a useful solvent and because it was identified that few studies evaluated nickel using water as a solvent [25] in general, and in the liquid phase, in particular. This reaction phase was defined in an attempt to reduce the energy cost associated with the reaction in vapor phase, due to the boiling temperature of levulinic acid (245 °C) [47,48]. It was also understood that using supports with acidic characteristics would favor the formation of γ-valerolactone; thus, titania was selected as the support.

## 2. Experimental Tests

### 2.1. Catalyst Synthesis

Titanium oxide presenting the anatase crystalline phase was synthesized via the sol-gel method by adding 12 mL of titanium isopropoxide (Aldrich, St. Louis, MO, USA) to 12.0 mL of 2-propanol (Merck, Darmstadt, Germany) under stirring followed by adding 6.0 mL of water. After 1 h of stirring, the solution was aged for 24 h, then dried at 80 °C for 6 h and calcined at 450 °C for 16 h. Ni was added to the titania support with a theoretical loading of 35 wt.% using the deposition–precipitation method via NH_3_ evaporation. First, 1.8 g of ammonium carbonate was dissolved in 20.0 mL of water and 20.0 mL of ammonium hydroxide solution (30%, Sigma, St. Louis, MO, USA), under stirring at ambient temperature. Second, 0.9216 g of nickel carbonate (Sigma) and 1.0 g of the TiO_2_ were added to the solution and heated to 80 °C for 3 h under a nitrogen atmosphere. After cooling to room temperature, the solid phase was extracted via centrifugation and washed with water until the pH was approximately 7, followed by drying at 80 °C overnight. Finally, Ni was reduced via a thermal treatment to 400 °C under a flow of H_2_ (50 mL/min, 10 °C/min heating rate). After 5 h at 400 °C, the catalyst was cooled and passivated using 1% O_2_/He (50 mL/min for 12 h). The sample was named Ni-TiO_2_-400.

Through prior experiments involving temperature-programmed reduction (TPR), BET physisorption, and in situ X-ray diffraction during the calcination and activation process, the optimal calcination temperature and reduction conditions for the catalysts was found. The aim was to stabilize support in a crystalline phase of TiO_2_ anatase and improve the reduction of Ni to the metal phase and the surface area. As a result, setting the calcination temperature to 450 °C and the activation step to 400 °C proved to be the most effective choices for enhancing the physical and chemical properties of the Ni-TiO_2_ catalysts.

### 2.2. Catalytic Reaction—Experimental

Table 1 exhibits the parameters considered in the reaction design. It is worth mentioning that the reaction parameters were determined based on a fractional factorial methodology with a central point. Also, experiments 3–5 are just triplicates of the same conditions.

The catalytic parameters, including temperature, hydrogen pressure, and catalyst loading, were derived from the literature [47,48]. Furthermore, it is widely recognized that non-noble metal catalysts (Ni) demand more thermal energy, higher loadings, and more significant hydrogen pressure to operate effectively in catalytic biomass conversion compared to noble catalysts such as Pt, Ru, and Ir. This work aims to explore the influence of these catalytic parameters to ascertain their potential and limitations concerning catalytic performance and kinetic mechanisms.

The catalytic reaction was carried out using a Hastelloy batch reactor (300 mL, Parr Instruments, Moline, IL, USA). An aqueous solution of levulinic acid (LA—35 g/L, Sigma) and 300 mg of catalyst were loaded into the reactor to give a total volume of 100 mL. The reactor was initially purged with N_2_ followed by H_2_ 3 times. Initially, the reactor was heated to 175 °C with a stirring speed of 50 rpm. As soon as the reaction temperature was reached, the stirring speed was raised to 800 rpm, and H_2_ was added to the reactor. Then, the initialization of the reaction was determined. H_2_ pressure was under isobaric conditions with pressures equal to 1.03, 2.07, or 3.10 psi. Aliquots from the reactor were taken for GVL and LA quantification at 0.5, 1, 2, 3, 4, and 5 to obtain points for the kinetic curve. The reaction products were identified and quantified with a gas chromatography (CG) Agilent model 7890A.

The catalytic parameters, conversion (*X*), product yield (*Y_GVL_*), and selectivity to GVL (*S_GVL_*_)_ were determined according to the equations:

(1) $$X_{t}=\frac{C_{LA,t_{0}}-C_{LA,t}}{C_{LA,t_{0}}}$$

(2) $$Y_{GVL,t}=\frac{C_{GVL,t}}{C_{LA,t_{0}}}$$

(3) $$S_{GVL,t}=\frac{Y_{GVL,t}}{X_{t}}$$

where $c_{LA,t_{0}}$, $c_{LA,t}$, and $c_{GVL,t}$ represent the initial concentration of the levulinic acid (mol·m^−3^), concentration of levulinic acid, and γ-valerolactone concentration in the aliquot removed from the reaction media in a specific time, respectively.

The results for conversion, product yield, and selectivity were presented in [49,50], for all the conditions tested. Therefore, they will not be repeated in this paper; only the main observations will be discussed, since the focus of this work is the kinetic model evaluation. However, those experimental results were used in the fitting of the parameters of the models proposed here.

A catalytic reaction was also conducted without levulinic acid to evaluate the catalyst’s stability under catalytic conditions. The catalyst aliquots tested were identical to those used in the catalytic protocols. Samples were collected at 0, 0.5, 1, 2, and 3 h throughout the experiment. The solid was separated through centrifugation, dried at 80 °C, and characterized using X-ray diffraction and Temperature-Programmed Oxidation (TPO). These techniques evaluate the catalysts’ crystalline structure and the oxidation state of nickel, indirectly indicating the metal’s reduction level. The results showed that titania remained in the anatase phase, nickel was present in its metallic form, and the degree of oxidation corresponded to the amount of metallic nickel in the activated catalysts before the catalytic reaction. These findings confirmed that the catalyst’s structural and activation parameters remained stable.

## 3. Kinetic Model

The hydrogenation of levulinic acid was carried out in the liquid phase, in the presence of a solid catalyst (nickel supported catalyst on titania). The route of reaction considered in this work can be represented by

HO-CO-CH_2_-CH_2_-CO-CH_3_ + H_2_ → HO-CO-CH_2_-CH_2_-CHOH-CH_3_ → C_5_H_8_O_2_ + H_2_O levulinic acid + hydrogen → 4-hydroxypentanoic acid → γ-valerolactone + water

### 3.1. Considerations About the Mechanism of Reaction

The catalyst studied in this work was nickel supported on anatase titanium dioxide. The route of reaction considered in this work had two main reactions: first, the hydrogenation of levulinic acid forming 4-hydroxypentanoic acid, and second, the dehydration (intramolecular esterification) of 4-hydroxypentanoic acid forming γ-valerolactone and water. The first reaction is favored by metallic sites, while the second reaction is favored by the acidity of the medium.

Results from the literature for different catalysts indicate that the adsorption of levulinic acid and hydrogen are not competitive, so that these molecules may be adsorbed by different sites and therefore may not compete for the same kind of site. For example, ref. [19] showed that for a Ru/C catalyst, the experimental data can be better explained by a kinetic model where the adsorption of levulinic acid and hydrogen take place at different metallic sites. On the other hand, ref. [15] showed that for a Ru/C catalyst, the model that best fitted to the experimental data was the one with competitive adsorption between levulinic acid, hydrogen, and γ-valerolactone.

Results specifically for the adsorption of levulinic acid over anatase titanium dioxide are more difficult to find in the literature. However, as indicated by [51], in general, carboxylic acids strongly adsorb to the surface of metallic oxides, forming a bond between the oxygen (in the carboxylate) and the cations in oxide surface. Also, ref. [52] proposed that, for a Cu–ZrO_2_ catalyst, hydrogen is adsorbed over the metallic copper surface, while the levulinic acid is adsorbed over the ZrO_2_ surface, and the reaction takes place in the interface of these two surfaces.

Therefore, in this work, which uses Ni–TiO_2_ as the catalyst, the kinetic model considers three different kinds of sites for adsorption. The first one (*l*_1_) is considered for the adsorption of levulinic acid (and so a noncompetitive adsorption between hydrogen and levulinic acid). As shown in the literature, this could be either a site in the metallic surface [15,19], or a site in the oxide surface [29,52]. In either case, independently of the characteristics of this kind of site, the mathematical shape of the equation for the rate of reaction is the same, using a Langmuir–Hinshelwood–Hougen–Watson (LHHW) kinetic model for heterogeneous catalytic reactions, but with a different physical meaning.

The second one (*l*_2_) is considered for the adsorption of hydrogen and has metallic characteristics (Ni^0^). Finally, the third kind of site (*l*_3_) is considered for the adsorption of γ-valerolactone and has acid characteristics (TiO_2_).

### 3.2. Proposed Mechanism for the Reactions

Since the reactions take place on the catalyst surface, the mechanism proposed here considered three different kinds of adsorption sites, leading to the following steps:

- adsorption–desorption:

LA + l_1_ ⇆ LA.l_1_ (4)

GVL + l_1_ ⇆ GVL.l_1_ (5)

HPA + l_1_ ⇆ HPA.l_1_ (6)

H_2_ + l_2_ ⇆ H_2_.l_2_ (7)

H_2_ + 2 l_2_ ⇆ 2 H.l_2_ (8)

AL + l_3_ ⇆ AL.l_3_ (9)

HPA + l_3_ ⇆ HPA.l_3_ (10)

GVL + l_3_ ⇆ GVL.l_3_ (11)

- reactions on the surface:

AL.l_1_ + H_2_.l_2_ ⇆ HPA.l_1_ + l_2_ (12)

AL.l_1_ + H.l_2_ ⇆ HAL.l_1_ + l_2_ (13)

HAL.l_1_ + H.l_2_ ⇆ HPA.l_1_ + l_2_ (14)

HPA.l_3_ ⇆ GVL.l_3_ (15)

where HPA is 4-hydroxypentanoic acid, HAL is an intermediate, and GVL is γ-valerolactone.

Using the proposed steps, 15 different models were proposed and tested for the hydrogenation of levulinic acid, depending on which step was considered as a determining step. Their rate of reactions is presented in Table 2 ($r_{M}$), considering different determining steps as controlling the rate of reaction, as follows:

▪ Adsorption of levulinic acid as the determining step.
▪ Adsorption of hydrogen as the determining step.
▪ Desorption of 4-hydroxypentanoic acid as the determining step.
▪ Reaction on the surface as the determining step.

The rate of formation of γ-valerolactone ($r_{A}$) is given by:

(16) $$r_{A}=k_{A}^{\prime }\cdot \frac{c_{HPA}-\frac{K_{GVL}^{A}}{K_{HPA}^{A}\cdot K_{sr}^{A}}\cdot c_{GVL}}{1+K_{LA}^{A}\cdot c_{LA}+K_{GVL}^{A}\cdot c_{GVL}+K_{HPA}^{A}\cdot c_{HPA}}$$

These models were then evaluated by statistical methods to verify which one provided the best fitting to experimental data.

## 4. Numerical Approach

The reactor was modeled as a BSTR (batch-stirred tank reactor), and the parameters of each kinetic model were fitted using the least-squares method. Since the stirring of the reactor was very good and the catalyst particles were very small, mass transfer resistance could be neglected in the model, so a uniform concentration for each species was considered through the position of the reactor, as well as the same concentration in the catalyst and the liquid medium for each species.

### 4.1. Mathematical Model

The model for the reactor is a BSTR with constant volume (V) at a constant temperature (T), where the variations of concentrations with time given by

(17) $$\frac{dc_{LA}}{dt}=-r_{M}\cdot \frac{m_{\mathrm{cat}}}{V}$$

(18) $$\frac{dc_{HPA}}{dt}=\left(r_{M}-r_{A}\right)\cdot \frac{m_{\mathrm{cat}}}{V}$$

(19) $$\frac{dc_{GVL}}{dt}=r_{A}\cdot \frac{m_{\mathrm{cat}}}{V}$$

where $c_{LA}$, $c_{HPA}$, and $c_{GVL}$ are the concentrations (gmol/m^3^) of levulinic acid, 4-hydroxypentanoic acid, and γ-valerolactone, respectively. The initial concentrations are zero (t=0). The rate of formation of γ-valerolactone is $r_{A}$ (Equation (16)), and the rate of hydrogenation of levulinic acid is $r_{M}$ (Table 2).

The concentration of hydrogen ($c_{H_{2}}$) was considered constant since the pressure of hydrogen was kept constant during each experiment. The value of $c_{H_{2}}$ is then calculated from $x_{H_{2}}$, given by Henry’s law, as follows:

(20) $$p_{H_{2}}=H\left(T\right)\cdot x_{H_{2}}\;$$

where Henry’s constant was calculated by [53]:

(21) $$A\cdot {\left(\log\;\overset{̿}{H}\right)}^{2}+B\cdot {\left(\frac{1}{\overset{̿}{T}}\right)}^{2}+C\cdot \frac{\log\overset{̿}{H}}{\overset{̿}{T}}+D\cdot \log\overset{̿}{H}+E\cdot \frac{1}{\bar{T}}-1=0$$

(22) $$\overset{̿}{T}=T\cdot 10^{-3}$$

(23) $$\overset{̿}{H}=H\cdot 10^{-4}$$

where the units are K for T and atm/mole fraction for H(T), and where A=−0.1233, B=−0.1366, C=0.02155, D=−0.2368, and E=0.8259 for hydrogen [53].

The concentration of hydrogen $c_{H_{2}}$ is calculated by multiplying the total molar concentration in the liquid phase $c_{L}$ by the molar fraction $x_{H_{2}}$. Since, in this work, $p_{H_{2}}$ was given in MPa, the following conversion was used:

(24) $$c_{H_{2}}=c_{L}\cdot \frac{1}{H\;\left[\mathrm{atm}/\mathrm{mole fraction}\;\right]}\cdot p_{H_{2}}\left[\mathrm{Mpa}\right]\cdot \frac{1\;\mathrm{atm}}{0.101325\;\mathrm{MPa}}$$

### 4.2. Fitting of the Kinetic Model Parameters

The fitting of the parameters was done using the least-squares method, minimizing the sum of square errors:

(25) $$S=\sum_{j=1}^{M}\sum_{i=1}^{N}e_{i,j}\cdot e_{i,j}^{T}=\sum_{j=1}^{M}\sum_{i=1}^{N}\left[{\hat{c}}_{i,j}-c_{i,j}\right]\cdot {\left[{\hat{c}}_{i,j}-c_{i,j}\right]}^{T}$$

where N is the number of catalytic tests, M is the number of aliquots sampled for each catalytic test, and ${\hat{c}}_{i,j}$ and $c_{i,j}$ are vectors for the calculated and experimental values of concentrations at each time $t_{i,j}$ for levulinic acid and γ-valerolactone.

### 4.3. Numerical Methods

The minimization problem was solved with python, using the software scipy, numpy, matplotlib, and lmfit, with the subroutine leastsq (Levenberg–Marquardt method). The system of ordinary differential equations was solved using the software odeint (solver LSODA).

## 5. Kinetic Model Evaluation

The experimental results indicated that γ-valerolactone is in equilibrium or near equilibrium with 4-hydroxypentanoic acid in the aliquots that were sampled, making it difficult to determine the kinetic parameters in the reaction (15). Since water is the solvent (almost constant concentration), the concentration of 4-hydroxypentanoic acid (HPA) can then be given by

(26) $$c_{HPA}=\frac{1}{K_{\mathrm{eq}}}\cdot c_{GVL}\;$$

so that the differential equations for the concentrations are only related with the rate of reaction in the metallic surface ($r_{M}$):

(27) $$\frac{dc_{LA}}{dt}=-r_{M}\cdot \frac{m_{\mathrm{cat}}}{V}$$

(28) $$\frac{dc_{HPA}}{dt}=\frac{1}{1+K_{\mathrm{eq}}}\cdot r_{M}\cdot \frac{m_{\mathrm{cat}}}{V}$$

(29) $$\frac{dc_{GVL}}{dt}=\frac{K_{\mathrm{eq}}}{1+K_{\mathrm{eq}}}\cdot r_{M}\cdot \frac{m_{\mathrm{cat}}}{V}$$

### 5.1. Evaluating the Kinetic Models

The first step was the fitting of each model to the experimental data obtained at 150 °C, 175 °C, and 200 °C. The Bartlett test was applied, and the results are presented in Table 3, Table 4 and Table 5. From these tables, only models 11 and 15 were considered adequate in the three data sets. After fitting the parameters of these two models, model 11 was selected as the most adequate. This model considered adsorption of hydrogen as non-dissociative and the reaction of hydrogenation of levulinic acid as the determinant step. Also, the fitting of the parameters indicated that the term associated with the adsorption of HPA was negligible in this model (see Table 2, model 11).

The fitted parameters for model 11 are presented in Table 6, where the equation for this model is given by

(30) $$r_{M}=k_{sr}^{\prime }\left(175\;{}^{\circ}C\right)\cdot \exp\left[-\frac{E_{A}}{R}\cdot \left(\frac{1}{T}-\frac{1}{448}\right)\right]\cdot \frac{c_{LA}\cdot c_{H_{2}}}{{\left(1+K_{LA}\cdot c_{LA}+K_{GVL}\cdot c_{GVL}+K_{H_{2}}\cdot c_{H_{2}}\right)}^{2}}$$

The data set for Table 6 were $p_{H_{2}}$= (1.03; 2.07; 3.10) MPa, $c_{H_{2}}$ = (169; 290; 410) mol/m^3^, and $K_{\mathrm{eq}}$ = 27.4.

### 5.2. Comparing with Other Studies

There are few studies in the literature that present the kinetics of hydrogenation of levulinic acid, usually with different catalysts, such as [29], using Ni-NiO, [15,19], using Ru/C, and [52] using Cu-ZrO_2_.

In general, the models that considered competitive adsorption had a better fitting to experimental data than the models that considered non-competitive adsorption for hydrogen and levulinic acid. That suggests that the active sites both for adsorption of levulinic acid and hydrogen correspond to the same kind of metallic site (Ni).

Comparing with other studies that used Ru/C [15,19], the activation energy found for Ni-TiO_2_ (47.0 kJ/mol) is slightly higher than the one found for Ru/C (43 a 45 kJ/mol), or similar, considering the confidence interval (Table 5). Comparing with the results for Cu-ZrO_2_ (68 kJ/mol) from other studies [52], the activation energy for Ni-TiO_2_ is significantly lower.

On the other hand, the activation energy determined for the Ni-TiO_2_ catalyst was lower than that determined for a 70 kJ/mol Ni-NiO catalyst; however, as mentioned in the literature review, due to the experimental conditions in these studies and the methodology to determine the activation energy, the activation energy determined would correspond to the esterification step of 4-hydroxypentanoic acid, while the activation energy determined for the Ni-TiO_2_ catalyst corresponds to the formation step of 4-hydroxypentanoic acid.

The only products determined in the catalytic tests were γ-valerolactone and 4-hydroxypentanoic acid; the yield of γ-valerolactone was limited by thermodynamic equilibrium. This would represent an advantage by reducing the costs associated with the γ-valerolactone separation/purification process. In terms of productivity, the best result was 0.053 mol GVL/g·h, obtained in the reaction at 200 °C, pressure of 2.3 MPa, and initial levulinic acid concentration of 169 mol/m^3^.

There are results superior to those reported in this work, for example, those shown by [25,26,28,36]. However, it is important to emphasize that the use of water as a solvent represents a challenge, due to the decreased stability of the catalysts and the loss of selectivity to γ-valerolactone. For example, in a solvent-free process, the Ni-MoOx/C catalyst has a productivity of 0.323 moles GVL/g·h, while it is deactivated when water is used as a solvent. In the case of another similar catalyst, Ni-Mo/C, when used in dioxane, showed 100% selectivity for γ-valerolactone, while when used with water, it preserves productivity (0.068 kJ/mol), but the selectivity for γ-valerolactone is decreased (84%). Therefore, the results obtained in the present study are relevant, from the point of view of productivity and selectivity to γ-valerolactone and the stability of the catalyst in aqueous medium.

Finally, the purpose of using titania as a support, in part, was to favor the esterification reaction of 4-hydroxypentanoic acid. However, it was not possible to determine kinetic parameters for this reaction, which suggests that the catalyst participates in this step of the reaction.

## 6. Conclusions

The prepared Ni-TiO_2_-400 catalysts are active and selective in the hydrogenation reaction of levulinic acid to γ-valerolactone in water using molecular hydrogen. Under the conditions applied in the catalytic reaction, the route of formation of γ-valerolactone is via 4-hydroxypentanoic acid. There was no evidence of side reactions. The 35Ni-TiO_2_-400 catalyst showed catalytic activity even at a temperature of 150 °C and high selectivity for γ-valerolactone (between 95% and 98%) in all conditions tested.

Hydrogen pressure contributed to the formation of γ-valerolactone, especially at low concentrations of levulinic acid; its effect on the hydrogenation rate is positive. However, at high concentrations of levulinic acid, a slight decrease the rate of hydrogenation was obtained.

The best fitting for the reaction kinetics was the one with a LHHW model in which the determining step was due to the reaction on the catalyst surface, with competitive adsorption between hydrogen, levulinic acid, and γ-valerolactone. Kinetic analyses using this model indicate an activation energy for the hydrogenation step of (47.0 ± 1.2) kJ·mol^−1^. The terms related to the adsorption on the metal surface of levulinic acid, γ-valerolactone, and 4-hydroxypentanoic acid were significant.

Under the reaction conditions, the esterification rate of 4-hydroxypentanoic acid was higher than the hydrogenation rate of levulinic acid and quickly reached equilibrium. This makes it difficult to obtain kinetic parameters for this reaction step.

Finally, there are good perspectives for using a titania-supported nickel catalyst, which is a non-noble catalyst, for the hydrogenation of levulinic acid to γ-valerolactone in water, using molecular hydrogen. In addition, the best model found in this work through a statistical analysis can be used for the design of such reactors.

## Figures and Tables

**Table 1:** Factorial experiment parameters applied in the LA to GVL catalytic reaction with Ni/TiO_2_ catalyst.

Experiment	H_2_ Pressure (MPa)	LA Initial Concentration (mol·m^−3^)	Temperature (°C)
1	2.07	169	175
2	1.03	290	175
3	2.07	290	175
4	2.07	290	175
5	2.07	290	175
6	2.07	410	175
7	3.10	290	175
8	3.10	410	200
9	1.03	410	200
10	1.03	169	200
11	2.07	290	200
12	3.10	169	200
13	1.03	169	150
14	3.10	410	150
15	3.10	169	150
16	1.03	410	150
17	2.07	290	150

**Table 2:** Rate of hydrogenation for levulinic acid ($r_{M}$).

Model	Rate Equation	Determining Step	Kind of Adsorption	Hydrogen Adsorption
1	$k_{LA}\cdot \frac{c_{LA}}{1+K_{GVL}\cdot c_{GVL}+K_{HPA}\cdot c_{HPA}+K_{H_{2}}\cdot c_{H_{2}}}$	04	C	M
2	$k_{LA}\cdot \frac{c_{LA}}{1+K_{GVL}\cdot c_{GVL}+K_{HPA}\cdot c_{HPA}+\sqrt{K_{H_{2}}\cdot c_{H_{2}}}}$	04	C	D
3	$k_{LA}\cdot \frac{c_{LA}}{1+K_{GVL}\cdot c_{GVL}+K_{HPA}\cdot c_{HPA}}$	04	NC	B
4	$k_{H_{2}}\cdot c_{H_{2}}$	07	NC	B
5	$\frac{k_{H_{2}}\cdot c_{H_{2}}}{1+K_{LA}\cdot c_{LA}+K_{GVL}\cdot c_{GVL}+K_{HPA}\cdot c_{HPA}}$	07	C	M
6	$\frac{k_{H_{2}}\cdot c_{H_{2}}}{{\left(1+K_{LA}\cdot c_{LA}+K_{GVL}\cdot c_{GVL}+K_{HPA}\cdot c_{HPA}\right)}^{2}}$	08	C	D
7	$k_{HPA}\cdot \frac{K_{sr}\cdot K_{LA}\cdot K_{H_{2}}\cdot c_{LA}\cdot c_{H_{2}}-K_{HPA}\cdot c_{HPA}}{1+K_{LA}\cdot c_{LA}+K_{GVL}\cdot c_{GVL}+K_{sr}\cdot K_{LA}\cdot K_{H_{2}}\cdot c_{LA}\cdot c_{H_{2}}}$	06	NC	B
8	$k_{HPA}\cdot \frac{K_{sr}\cdot K_{LA}\cdot K_{H_{2}}\cdot c_{LA}\cdot c_{H_{2}}-K_{HPA}\cdot c_{HPA}}{1+K_{LA}\cdot c_{LA}+K_{GVL}\cdot c_{GVL}+K_{sr}\cdot K_{LA}\cdot K_{H_{2}}\cdot c_{LA}\cdot c_{H_{2}}+\sqrt{K_{H_{2}}\cdot c_{H_{2}}}}$	06	C	D
9	$k_{HPA}\cdot \frac{K_{sr}\cdot K_{LA}\cdot K_{H_{2}}\cdot c_{LA}\cdot c_{H_{2}}-K_{HPA}\cdot c_{HPA}}{1+K_{LA}\cdot c_{LA}+K_{GVL}\cdot c_{GVL}+K_{sr}\cdot K_{LA}\cdot K_{H_{2}}\cdot c_{LA}\cdot c_{H_{2}}+K_{H_{2}}\cdot c_{H_{2}}}$	06	C	M
10	$k_{sr}\cdot \frac{K_{LA}\cdot K_{H_{2}}\cdot c_{LA}\cdot c_{H_{2}}-\frac{K_{HPA}}{K_{sr}}\cdot c_{HPA}}{\left(1+K_{LA}\cdot c_{LA}+K_{HPA}\cdot c_{HPA}+K_{GVL}\cdot c_{GVL}\right)\cdot \left(1+K_{H_{2}}\cdot c_{H_{2}}\right)}$	12	NC	M
11	$k_{sr}\cdot \frac{K_{LA}\cdot K_{H_{2}}\cdot c_{LA}\cdot c_{H_{2}}-\frac{K_{HPA}}{K_{sr}}\cdot c_{HPA}}{{\left(1+K_{LA}\cdot c_{LA}+K_{HPA}\cdot c_{HPA}+K_{GVL}\cdot c_{GVL}+K_{H_{2}}\cdot c_{H_{2}}\right)}^{2}}$	12	C	M
12	$k_{{sr}_{1}}\cdot \frac{K_{LA}\cdot \sqrt{K_{H_{2}}}\cdot c_{LA}\cdot \sqrt{c_{H_{2}}}}{\left(1+K_{LA}\cdot c_{LA}+K_{HPA}\cdot c_{HPA}+K_{GVL}\cdot c_{GVL}\right)\cdot \left(1+\sqrt{K_{H_{2}}\cdot c_{H_{2}}}\right)}$	13	NC	D
13	$k_{{sr}_{1}}\cdot \frac{K_{LA}\cdot \sqrt{K_{H_{2}}}\cdot c_{LA}\cdot \sqrt{c_{H_{2}}}}{{\left(1+K_{LA}\cdot c_{LA}+K_{HPA}\cdot c_{HPA}+K_{GVL}\cdot c_{GVL}+\sqrt{K_{H_{2}}\cdot c_{H_{2}}}\right)}^{2}}$	13	C	D
14	$k_{{sr}_{2}}\cdot \frac{K_{{sr}_{1}}\cdot \left(K_{LA}\cdot K_{H_{2}}\cdot c_{LA}\cdot c_{H_{2}}-\frac{K_{HPA}}{K_{sr}}\cdot c_{HPA}\right)}{\left(1+K_{LA}\cdot c_{LA}+K_{HPA}\cdot c_{HPA}+K_{GVL}\cdot c_{GVL}+K_{sr1}\cdot K_{LA}\cdot \sqrt{K_{H_{2}}}\cdot c_{LA}\cdot \sqrt{c_{H_{2}}}\right)\cdot \left(1+\sqrt{K_{H_{2}}\cdot c_{H_{2}}}\right)}$	14	NC	D
15	$k_{{sr}_{2}}\cdot \frac{K_{{sr}_{1}}\cdot \left(K_{LA}\cdot K_{H_{2}}\cdot c_{LA}\cdot c_{H_{2}}-\frac{K_{HPA}}{K_{sr}}\cdot c_{HPA}\right)}{{\left(1+K_{LA}\cdot c_{LA}+K_{HPA}\cdot c_{HPA}+K_{GVL}\cdot c_{GVL}+\sqrt{K_{H_{2}}\cdot c_{H_{2}}}+K_{{sr}_{1}}\cdot K_{LA}\cdot \sqrt{K_{H_{2}}}\cdot c_{LA}\cdot \sqrt{c_{H_{2}}}\right)}^{2}}$	14	C	D

C = Competitive; NC = Non-competitive; M = Molecular; D = Dissociative; B = Both.

**Table 3:** Bartlett test for models fitted to data at 150 °C.

Iteration	$\chi_{\mathrm{data}}^{2}$	$\chi_{\mathrm{tab}}^{2}$	Model Eliminated	${\hat{\sigma }}_{\varepsilon }^{2}$ Model Eliminated
01	679.0	23.7	1	188.1
02	597.3	22.4	2	186.9
03	474.5	21.0	3	183.5
04	257.2	19.7	7	47.6
05	230.2	18.3	4	43.5
06	197.5	16.9	14	33.1
07	176.5	15.5	10	25.3
08	164.3	14.1	8	24.6
09	148.3	12.6	9	23.5
10	126.3	11.1	12	22.1
11	90.6	9.5	13	12.8
12	75.6	7.8	6	10.2
13	59.8	6.0	5	10.0
14	0.2	3.8	–	–

**Table 4:** Bartlett test for models fitted to data at 175 °C.

Iteration	$\chi_{\mathrm{data}}^{2}$	$\chi_{\mathrm{tab}}^{2}$	Model Eliminated	${\hat{\sigma }}_{\varepsilon }^{2}$ Model Eliminated
01	604.9	23.7	4	252.5
02	566.2	22.4	2	241.7
03	521.4	21.0	1	236.5
04	460.8	19.7	3	235.4
05	365.9	18.3	6	214.7
06	221.1	16.9	5	167.1
07	6.8	15.5	–	–

**Table 5:** Bartlett test for models fitted to data at 200 °C.

Iteration	$\chi_{\mathrm{data}}^{2}$	$\chi_{\mathrm{tab}}^{2}$	Model Eliminated	${\hat{\sigma }}_{\varepsilon }^{2}$ Model Eliminated
01	1633.4	23.7	5	7397.3
02	1354.9	22.4	4	5004.2
03	986.4	21.0	6	2647.3
04	597.3	19.7	2	609.4
05	508.2	18.3	1	603.4
06	350.6	16.9	3	535.0
07	8.9	15.5	–	–

**Table 6:** Fitted parameters for model 11.

Parameter	Value	Bounds (95% Interval)		Units
		Lower	Upper	
$K_{LA}$	12.6	11.3	14.4	dm^3^/mol
$K_{H_{2}}$	20.0	17.3	23.4	dm^3^/mol
$k_{sr}^{\prime }(175\;{}^{\circ}C)$	32.5	26.7	40.5	dm^6^/mol·g_cat_·h
$E_{A}$	47.0	45.7	48.2	kJ/mol
$K_{GVL}$	2.1	1.5	2.8	dm^3^/mol

## Data Availability

Data is contained within the article.

## List of Symbols

$c_{i}$	concentration of species i in the liquid phase (mol/m^3^)
$K_{i}$	adsorption equilibrium constant for species i
$m_{\mathrm{cat}}$	catalyst mass (g)
P	pressure (MPa)
$r_{k}$	rate of reaction k
S	sum of square errors
$S_{i}$	selectivity for product i
t	time
T	temperature (K)
$x_{H_{2}}$	molar fraction of hydrogen in the liquid phase
X	conversion
$Y_{i}$	product yield
*Subscripts*	
GVL	γ-valerolactone
LA	levulinic acid
HPA	4-hydroxypentanoic acid
